# Novel Biodegradable Porous Scaffold Applied to Skin Regeneration

**DOI:** 10.1371/journal.pone.0056330

**Published:** 2013-06-10

**Authors:** Hui-Min Wang, Yi-Ting Chou, Zhi-Hong Wen, Zhao-Ren Wang, Chun-Hong Chen, Mei-Ling Ho

**Affiliations:** 1 Department of Fragrance and Cosmetic Science, Kaohsiung Medical University, Kaohsiung, Taiwan; 2 Graduate Institute of Natural Products, Kaohsiung Medical University, Kaohsiung, Taiwan; 3 Department of Physiology, College of Medicine, Kaohsiung Medical University, Kaohsiung, Taiwan; 4 Department of Marine Biotechnology and Resources, Asia-Pacific Ocean Research Center, National Sun Yat-sen University, Kaohsiung, Taiwan; 5 Orthopaedic Research Center, Kaohsiung Medical University, Kaohsiung, Taiwan; University Hospital Hamburg-Eppendorf, Germany

## Abstract

Skin wound healing is an important lifesaving issue for massive lesions. A novel porous scaffold with collagen, hyaluronic acid and gelatin was developed for skin wound repair. The swelling ratio of this developed scaffold was assayed by water absorption capacity and showed a value of over 20 g water/g dried scaffold. The scaffold was then degraded in time- and dose-dependent manners by three enzymes: lysozyme, hyaluronidase and collagenase I. The average pore diameter of the scaffold was 132.5±8.4 µm measured from SEM images. With human skin cells growing for 7 days, the SEM images showed surface fractures on the scaffold due to enzymatic digestion, indicating the biodegradable properties of this scaffold. To simulate skin distribution, the human epidermal keratinocytes, melanocytes and dermal fibroblasts were seeded on the porous scaffold and the cross-section immunofluorescent staining demonstrated normal human skin layer distributions. The collagen amount was also quantified after skin cells seeding and presented an amount 50% higher than those seeded on culture wells. The *in vivo* histological results showed that the scaffold ameliorated wound healing, including decreasing neutrophil infiltrates and thickening newly generated skin compared to the group without treatments.

## Introduction

Skin, the largest organ in the body of vertebrates, is composed of the epidermis, dermis and hypodermis [1]. It plays a significant role in preventing the body from many chemical or mechanical damages [2]. Severe acute and chronic wounds on the skin such as burns, abrasions, lesions, or leg ulcers result in a substantial loss of dermal tissues that poses great challenges to the healing process. Because of the antigenicity of donor tissue and the limitation of donor sources, skin grafts cannot achieve complete skin recovery and this makes it unable to be used widely [3]–[7]. It is an essential issue to enhance skin cell growth during wound healing so in recent years, several models for skin epidermis and dermis reconstruction have been developed [8]–[10]. A number of experimental studies deal with new approaches to improve human skin cell growth using either physical, pharmacological or phytotherapeutic methods [11]–[13]. Tissue engineering is an effective method for developing skin substitutes and ameliorating the healing process. In tissue engineering, one vital aim of cell biologists is to stimulate cell proliferation using sophisticated culturing conditions. This study aimed to develop a scaffold that improve cell growth and imitate the structure of the skin epidermis and dermis.

Collagen is an essential constituent and major component of human connective tissues, especially in skin soft tissues [14]. For many years, porous collagen scaffolds have been widely used in tissue engineering for skin, cartilage, bone and nerve [15]. Collagen is widely used for skin tissue engineering and coating materials and it is known to be one of the most promising biomaterials for diverse applications [16], [17]. Advantages include good biocompatibility, low toxicity and controllable biodegradability in addition to well-documented bio-structural, physical, chemical and immunological properties [18]–[22]. Despite the advantages, the weak mechanical strength and fast biodegradation rate of uncross-linked collagen scaffolds are the critical problems that limit its applications. Hence, cross-linking the collagen-based scaffolds is an efficient manner to optimize the mechanical property and to adjust the biodegradation rate. Hyaluronic acid (HA) is another main component of the skin that is associated with tissue repair. Being the most hygroscopic molecule present in nature and in the skin, HA's characteristic is of fundamental importance in water retention [23]. HA can be further modified by hydroxyl and carboxyl functional groups with certain cells or with extracellular matrix (ECM) components to give its biological functions [24]. Also, HA plays comprehensive functions in the mediation of skin-cell biological processes and matrix events, cell migration, proliferation, granulation tissue matrix organization, inflammatory response moderation, re-epithelialization and scarring [25]. In addition, gelatin, denatured collagen promotes cellular attachment, growth, and differentiation. Gelatin is an inexpensive and non-immunogenic protein component with well-defined physical, chemical, biological properties that has been widely used in a variety of skin wound dressings. In general, these three components are easily degraded, resulting in a loss of mechanical strength that makes it difficult to maintain bio-structures [26]. Accordingly, in this work, we hypothesize that cross-linking these three materials may modify the degradation rate and biomechanical characteristics, thus improving their biocompatibility.

The cross-linking of bio-scaffolds has become one of the leading strategies for the bio-porous matrix. Currently, there are two kinds of cross-linking methods frequently employed in improving the mechanical properties: physical treatments and chemical techniques [27], [28]. Physical treatments usually cannot yield a high enough cross-linking degree to meet the demands for mechanical strength and biodegradation rates, therefore, treatments by chemical methods are still necessary in most cases [29], [30]. A cross-linking agent, ethyl-3-(3-dimethylaminopropyl) carbodiimide hydrochloride (EDC), is of great interest in maximizing the extent of cross-linking because it contains 2 different reactive groups that are able to directly link 2 various amino acid side chains, and it is a zero-length cross-linking agent (i.e., the agent itself is not incorporated into the macromolecule) [31]. Therefore, in the present study, we used EDC to cross-link collagen, HA and gelatin together constructing a collagen/HA/gelatin scaffold. This porous sponge-like scaffold was tested by *in vitro* bio-functional assays and was co-cultured with keratinocytes (KCs), melanocytes (MCs) and fibroblasts (FBs). We further examined its dressing properties in wound healing on rat skin *in vivo*.

## Materials and Methods

### Manufacturing collagen/HA/gelatin sponge bio-porous scaffold

**Figure 1:**
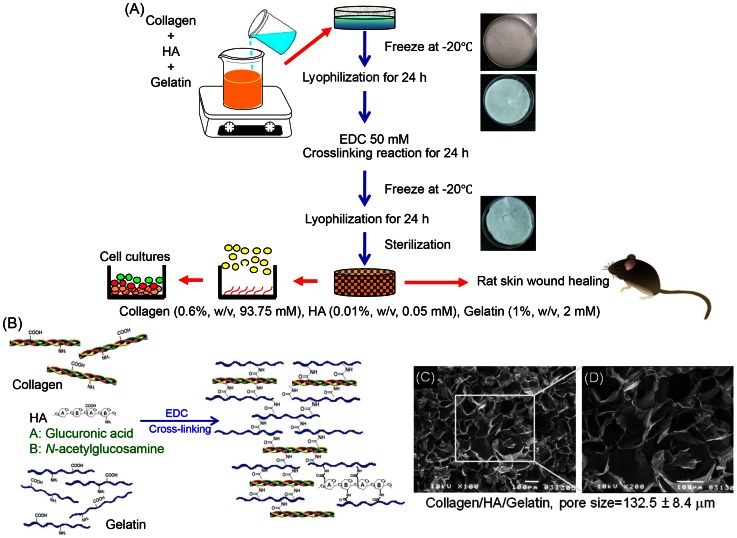
The manufacturing process and structural diagram. (A) The biomaterial manufacture, skin culture and mouse skin wound healing model. (B) Proposed schematic presentation of collagen (0.6%, w/v, 93.75 mM), HA (0.01%, w/v, 0.05 mM), gelatin (1%, w/v, 2 mM) cross-linked by EDC. (C), (D) SEM of collagen/HA/gelatin scaffolds. The pore size was 132.5±8.4 μm.

Collagen (MW: 64,000), gelatin (MW: 5,000) and EDC were all purchased from Sigma-Aldrich Chemical (St. Louis, USA). HA (MW: 2–2.1 MDa) was obtained from Kibun Food Chemicals (Tokyo, Japan). A solution of collagen, HA and gelatin were mixed with final concentrations of 93.75 μM, 0.05 μM, and 2 mM, respectively. The mixed solution was poured gently into a 6-cm petri dish and frozen at −20°C for 24 h. The scaffold size could be regulated by controlling the working volume of the fabricating solution. The collagen/HA/gelatin sponge was constructed by lyophilizing the frozen solution for 24 h. It was then chemically cross-linked for 24 h at 25°C in pure ethanol containing 50 mM EDC. Afterwards, the reaction was terminated by removing the EDC solution and washed with distilled H_2_O for more three times remov un-reacted chemicals. The scaffold was lyophilized for another 24 h and sterilized by ethylene oxide gas. The dried collagen/HA/gelatin scaffold was manufactured in suitable size by controlling the working volume for further research. Figure 1A and B was drawn according to reactions derived from the established reaction paths in homogeneous phase.

### Scanning electron microscopy (SEM)

The morphological characteristics of collagen/HA/gelatin porous scaffolds were observed by using SEM (JEOL, Tokyo, Japan) (Figure 1C). Scaffolds were fixed in 2.5% glutaraldehyde in 0.1 M sodium phosphate buffer, pH 7.2 overnight, and post-fixed in 1% osmium tetroxide for 1 h, dehydrated in ethanol and critical-point dried. Dried samples were coated with gold via a sputter-coater at ambient temperature. Micrographs of scaffolds were taken and the pore size distribution was determined using Beckman Coulter LS32 equipment with a range of 0.01 µm to 1,000 µm. The average pore size was calculated by measuring the pore size of 30 pores on each of the 6 SEM photos.

### Swelling ratio assay

The water absorption for the medium diffusion is necessary to cells gaining essential nutritions. In general, all three components we used have a large number of negatively charged carboxylic groups within their backbone, and are hydrophilic adsorbing water. 30 mg scaffold samples were separately immersed into distilled water at 25°C for 4 h. After removal from the water, scaffolds were hung up until no dripping water was observed and then weighed. The absorption of water within the swollen scaffold was calculated by the following equation:
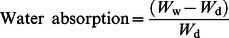
(1)where *W*_w_ is the weight of the swollen scaffold, and *W*_d_ is the weight of the dry scaffold. The swelling results were also compared with four other commercial wound dressings.

### Mechanical properties of the collagen/HA/gelatine-scaffold

The hardness assessments were performed with GS-709N (type A, Teclock Durometer, Japan), and the testing scaffolds were weighed at 30 mg. The value of the scaffold hardness is 58 (kgf/cm^2^). The melting profile of the scaffold was identified with a 2010 Differential Scanning Calorimeter (2010 DSC, TA Instruments Inc., New Castle, DE). The testing scaffolds were weighed (30 mg), and an empty aluminum pan was used as a reference. The samples were heated to 200°C and held for 15 minutes to gain the melting profile. The thermogram was analyzed and performed with Universal Analysis 2000 (TA Instruments Inc., New Castle, DE). The scaffold melting temperature is 101.35°C.

### *In vitro* enzyme degradation: lysozyme, hyaluronidase and collagenase I

Lysozyme is a glycoside hydrolase that catalyzes the hydrolysis of 1,4-β-linkages between *N*-acetylmuramic acid and *N*-acetyl-D-glucosamine residues or between *N*-acetyl-*D*-glucosamine residues. Hyaluronidase degrades the dermal ECM surroundings by hydrolysis and it plays an important role in ECM metabolism. Collagenase cleaves collagen fibrils at a precise site (Gly_775_–Leu/Ile_776_), which is located in the region with a relatively loose triple helical structure domain. Dry scaffolds (30 mg) immersed in 1 ml of phosphate-buffered saline (PBS buffer, pH 7.4, BioWest Co.) with lysozyme (10,000 and 30,000 U/ml) for 1, 3, 5, and 7 days, respectively, to examine the scaffold degradation rate. Similar protocols were applied to hyaluronidase, and the scaffolds was suspended in PBS (pH 7.4) containing 30 and 50 U/ml hyaluronidase for various time periods. For collagenase biodegradation tests, 1 ml of 0.1 M Tris–HCl with 0.05 M CaCl_2_ (pH 7.4) containing 10 and 20 U/ml collagenase I was used. Three enzymes were from Sigma-Aldrich Co. All reactions were incubated at 37°C, and a total of 0.2 ml of 0.25 M ethylenediamine tetraacetic acid (EDTA) was added to terminate the digestion after specific time intervals. At the end of testing periods, the remaining scaffolds were washed three times in distilled water and finally lyophilized for the following weight measurements. Degradation rates were determined from the weight of residual scaffolds and expressed as a percentage of the original weight.

### Human primary skin keratinocytes, melanocytes and fibroblasts cultures

Human KCs, MCs and FBs were cultured as modified methods from previous reports [32], [33]. KCs were isolated from foreskin primary culture, which were obtained from Chung-Ho Memorial Hospital, Kaohsiung Medical University, Taiwan (KMUH–IRB–990269). Human KCs were cultured in Keratinocyte-SFM (GIBCO™), supplemented with Bovine Pituitary Extract (BPE), and EGF Human Recombinant. The medium and growth supplement for KCs contains γ-epidermal growth factor, BPE, insulin, fibroblast growth factor and calcium (0.09 mM). Neonatal foreskin primary human epidermal MCs (HEMn-MP) were purchased from Cascade Biologics^TM^ and cultured in a melanocyte growth medium (Cascade M254–500). Medium 254 is a basal medium containing essential and non-essential amino acids, vitamins, organic compounds, trace minerals, and inorganic salts. The human melanocyte growth supplement contains bovine pituitary extract, fetal bovine serum, bovine insulin, bovine transferrin, basic fibroblast growth factor, hydrocortisone, heparin, and phorbol 12-myristate 13-acetate. The FBs were from KMUH as above [32], [33]. All types of cells were incubated at 37°C in a humidified incubator with 5% CO_2_ atmosphere.

### Human skin cell cultures in plates or porous scaffold

The scaffolds were sterilized with ethylene oxide gas, pre-wetted to exclude the remaining ethylene oxide and placed in 24-well plates. The appropriate volume of 100 μl cell suspension (5×10^5^ cells/100 μl) was loaded onto the top surface of each pre-wetted scaffold. The cells/scaffold constructs were then incubated at 37°C under 5% CO_2_ conditions for 4 h for the cells penetrating into the scaffold and adhere. After cell adherence, the cells/scaffold constructs were transferred to a new 24-well plate in order to remove the lost cells at the bottom of the wells, and 0.5 ml of culture medium was added in each new well. The medium was changed every other day. At every indicated time interval, the constructs were collected for further experimental analysis.

A human skin co-culture model was generated by seeding 10^6^ KCs and 10^5^ MCs on the scaffold that had been pre-seeded 7 days in advance with 5×10^5^ FBs using FBs medium. After KCs and MCs were seeded, the cells were incubated for another 7 days. During co-culture, we mixed the culture mediums with the same ratio to cell type amounts.

### 3-(4,5-dimethylthiazol-2-yl)-5-(3-carboxymethoxyphenyl)-2-(4-sulfophenyl)-2H-tetrazolium (MTS) assay

To study the cell viabilities and proliferation rate, we used MTS assay [34]. MTS (in the presence of phenazine methosulfate) produces a formazan product that has an absorbance maximum at 490–500 nm in phosphate-buffered saline solution. MTS assay was taken up by live cells, reduced by the dehydrogenase enzymes and released back into the culture medium as a yellow formazan product. This method was not influenced by the background fluorescence. The amount of formazan product, measured by absorbance at 490 nm, is directly proportional to the number of living cells in the culture. The cells in 100 μl medium were exposed to 20 μl of CellTiter 96 AQueous One Solution (Promega, USA) for 3 h according to the manufacturer instructions. Absorbance at 490 nm was recorded using a spectrometer plate reader (UV-vis, BioTek, USA).

### PKH-67 labeling

Cell pellets were mixed well with 5 μM PKH-67 (a green fluorescent dye that incorporated aliphatic reporter molecules into the cell membrane by selective partitioning; Sigma-Aldrich, USA) and incubated for 5 min at 25°C and gently vortexed every 30 s (according to the manufacturer protocols). Unincorporated PKH-67 was removed by washing the cells with complete medium. Monolayer PKH-67 labeled cells were re-seeded into the scaffold at a density of 1×10^5^/μl, and then harvested to take fluorescent images, respectively.

### Immunofluorescence analysis

We followed published protocols with minor modifications [11], [35]. Specimens of the cell seeded scaffold were fixed with 4% formaldehyde prepared in PBS for 24 h at room temperature. The specimens were embedded in paraffin, and cut into 5 μm sections. Sections were de-waxed, then permeabilized with 3% H_2_O_2_ in PBS for 15 min at room temperature, followed by blocking with FBS for one hour, and then incubated with primary antibodies against cytokeratin (for KCs) or s-100 (for MCs). Sections were then washed and treated with cy3-conjugated goat anti-rabbit antibody (Millipore) and FITC-conjugated goat anti-mouse antibody (Millipore) for 30 min at 25°C and counterstained with 4,6-diamidino-2-phenylindole (DAPI) (Vector, Burlingame, CA). Immunofluorescence images were taken (TE300; Nikon, Japan). Sections were also stained with hematoxylin and eosin (H&E) stain to check the localization of human skin cells.

### Collagen quantification

To measure the total collagen amount secreted by cells seeded in the scaffold, Sirius Red dye (Sigma-Aldrich, USA) was used to stain collagen [36]. We compared the collagen secreted by FBs, KCs, and MCs co-cultured on well surface directly or in the scaffold. 10^5^ FBs seeded on the 48-well plate or in the scaffold, and after 7 days, KCs and MCs were seeded in for another 7 days. After indicated time interval, the medium was removed and the cells were washed with PBS twice. 100 μl of 0.1% Sirius Red stain (0.05 g Sirius red powder per 50 ml picric acid) was added to each well and kept at 25°C for 1 h. The unattached stain was removed and washed for five times with 200 μl of 0.1 N HCl. The attached stain was extracted with 100 μl of 0.1 N NaOH (15 min) and mixed well to read the absorbance at 540 nm. By quantifying the collagen amount of the scaffold without cell seeding and deducting the OD value, the collagen/HA/gelatin scaffold background was excluded. To evaluate the specific collagen amount of each cell, the total collagen amount was divided by the total cell numbers.

### Animal experiments

In this study, the use of animals complied with the Guiding Principles in the Care and Use of Animals of the American Physiology Society and was approved by the National Sun Yat-sen University and Use Committee. Surgery and photographs were performed under isoflurane anesthesia. Male Wistar rats (250–285 g) were used for all experiments in this study. The rats were housed in Plexiglas cages in a temperature-controlled room (22±1°C), on a 12-hour/12-hour light/dark schedule, and with free access to food and water. Twelve rats were randomly divided into 2 groups, skin injury and scaffold treatment groups. The excision wound healing test was modified from Huang & Yang [37]. Following anesthetizing, dorsal hair was shaved by electric razor, a full thickness excisions of 2 cm in diameter were created with a surgical knife. After a back skin excision was made, a scaffold of equal size was rinsed by saline and covered on the wound immediately. In the injury group, wounds were not covered as a comparison. After surgery, rats were placed in individual cages for recovery.

### Evaluation of the wound size

Photographs were taken at the 1, 2, 3, 4, 5, 7 and 10 days after injury using a digital camera (Coolpix P6000, Nikon, Japan) with protocol parameters (F7.2, 1/60). The dressing on the wound was not detached during healing process unless the substance could be removed easily by sterilized cotton swabs. SPOT software (Diagnostic Instruments, Inc., Sterling Heights, MI, USA) was used to examine the area of each skin wound. There were some random sani chips on the wound as seen on the photos which would not affect the wound size measurements. The degree of wound healing was expressed as the percentage of wound area, calculated as
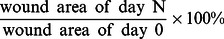
(2)


### Histological study

Ten days after injury, rats were sacrificed by over-dose anesthesia. Wound skin was fixed in 4% paraformaldehyde. The skin was then stained with hematoxylin and eosin (H&E) for histological observation. For histological analysis, images were captured with a Spot Xplorer CCD integrating camera (Diagnostic Instruments, Inc., Sterling Heights, MI, USA) using a Leica DM-6000 microscope (Leica, Wetzlar, Germany). The histological analysis was modified from Bayat *et al*. [38]. 10 randomly selected areas of dermis from each sample were examined at a magnification of 400× in order to count neutrophils. Histological examinations were performed in a blind fashion.

### Statistical analysis

All data were presented as the mean ± standard error. For statistical analysis, all data were analyzed with one-way analysis of variance (ANOVA) followed by the Duncan's method for multiple comparisons. A significant difference was defined as *p*<0.05.

## Results

### Morphology of porous scaffold (SEM image)

The thickness of the scaffold in this study was about 1 mm to mimic the real thickness of human skin including the epidermis and dermis. The SEM observation results of Figure 1C and D showed the morphological characteristics of the collagen/HA/gelatin scaffolds. The scaffold revealed highly interconnected porous structures, and the pore wall surface appeared smooth and homogeneous. SEM image of the sponge-like scaffold indicated that it had open macro-porous structures with the pore size of 132.5±8.4 μm.

### Water absorption ability

**Figure 2:**
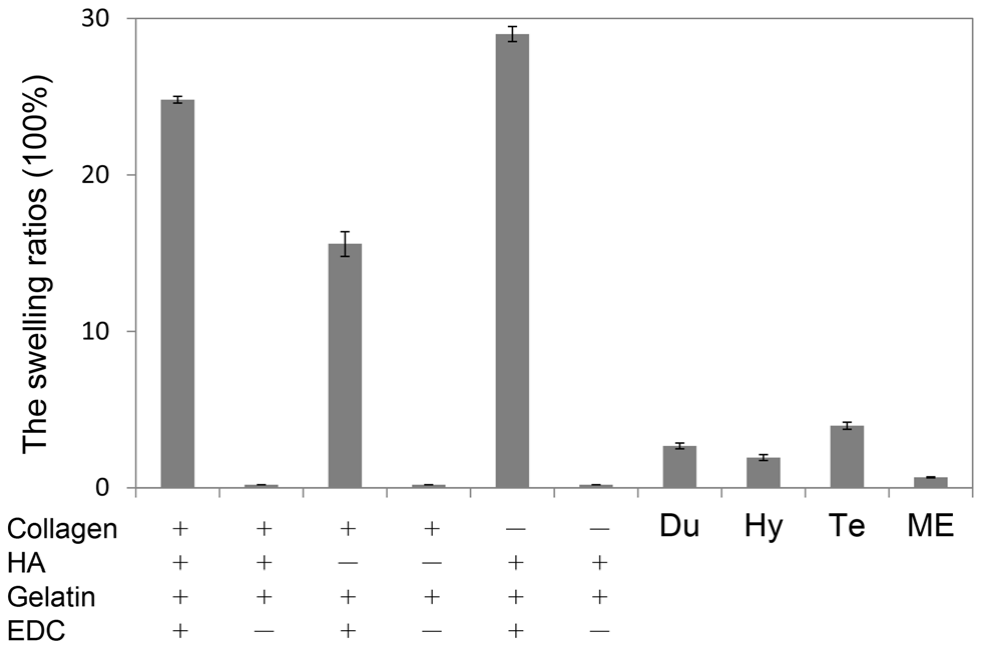
The swelling studies of the scaffolds fabricated with collagen (0.6%, w/v), HA (0.01%, w/v), and gelatin (1.0%, w/v) and crosslinked with EDC (50 mM) (n = 3). Without EDC crosslinking reactions, the scaffolds were dissoluble into water (symbol: ×). Compared with commercial materials include, Du (DuoDERM 9C52552), Hy (Hydro Coll), Te (Tegaderm M1635), and ME (MEDPOR^®^).

We used the swelling ratio test to evaluate water absorption abilities and showed in Figure 2. Without EDC cross-linking, scaffolds were dissolved into water within 5 minutes and couldn't sustain solid constructions. Our three raw components cross-linked with EDC presented a swelling ratio of more than 20 g water/g dried scaffold. Without HA, the swelling ratio value was decreased by 15, which indicated that HA was a significant factor with high water containing ability which is consistent with common knowledge. We changed the concentrations of three materials for cross-linking, including elevating and reducing, and the data didn't show obvious changes in the water absorption abilities (data not shown). Four commercial skin dressings were also examined for comparing and all swelling ratios were less than 10 folds.

### Scaffold degradation rates by lysozyme, hyaluronidase and collagenase I

**Figure 3:**
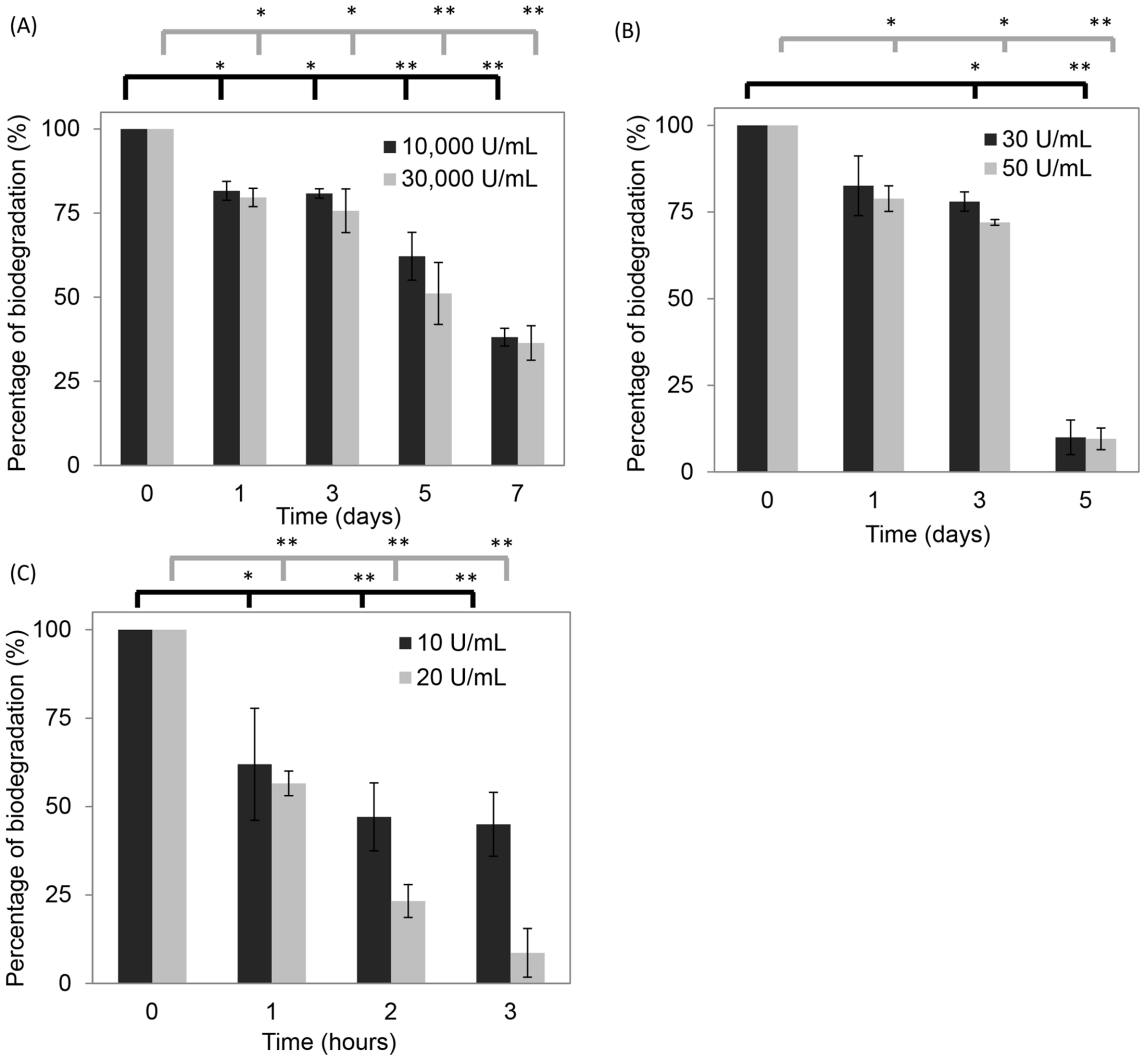
The degradation rates of the enzymes. (A) Lysozyme, (B) hyaluronidase, and (C) collagenase. A significant difference compare to the control group was defined as *p<0.05 and **p<0.01.

The biological degradation of the collagen/HA/gelatin sponge-like scaffold was determined by measuring the decrease in weight. The rates were tested by *in vitro* enzyme assays of lysozyme, hyaluronidase or collagenase I. Figure 3A showed that both 10,000 and 30,000 U/ml of lysozyme decomposed the scaffold gradually in one week. The scaffold was 38.1±2.6% and 36.4±5.1% of original weight after 7 day treatments, respectively. From Figure 3B, with 30 U/ml of hyaluronidase, the scaffold retained about 10.0% after a 5-day examination. With 50 U/ml of hyaluronidase, the scaffold was degraded after 7 days. Immersed in 20 U/ml collagenase I for 3 h, the scaffold was almost degraded (Figure 3C), and compared to 10 U/ml, the scaffold remained about 45.0% of the original weight. All three *in vitro* enzyme biodegradations were assessed to show time- and dose-dependences of this scaffold.

### Cell proliferation rate

**Figure 4:**
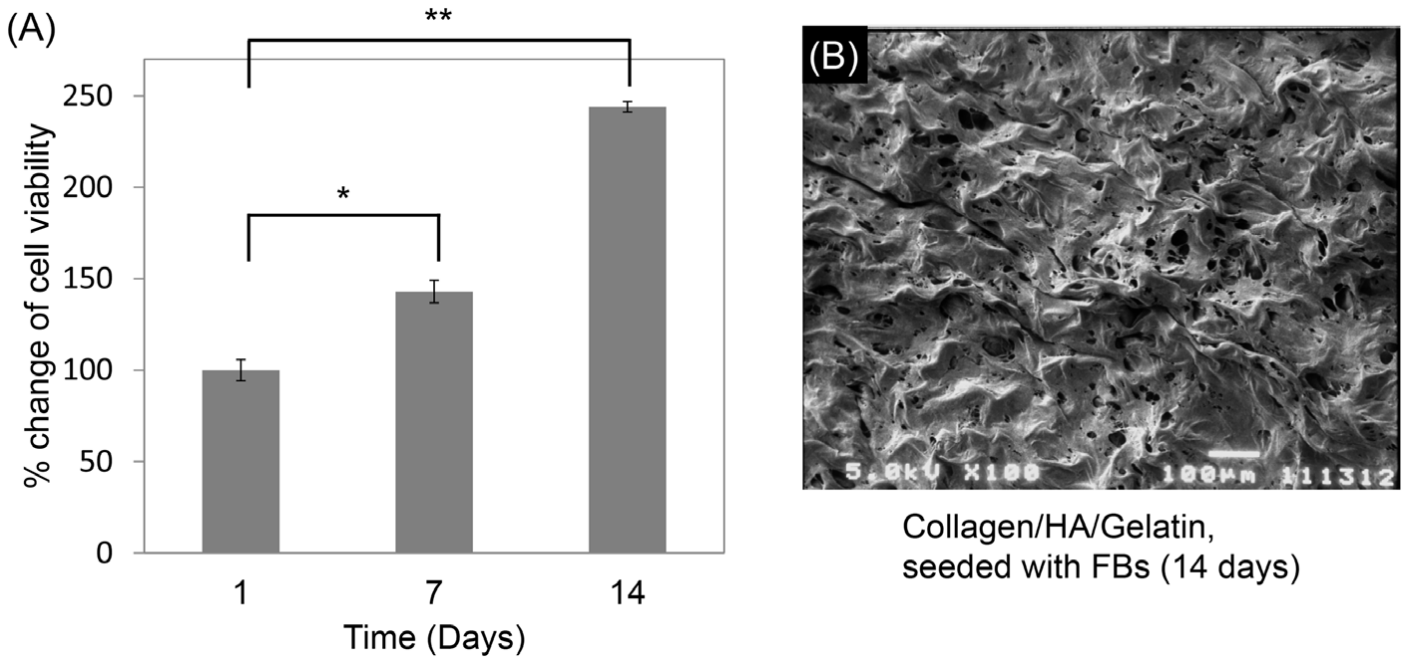
Cell proliferation ratios of human skin FBs seeded in the scaffolds (n = 4). From 1 to 14 days, the proliferation rate was observed by MTT assay (A). The SEM image of FBs seeding in scaffold for 14 days (B).

To assure the cytocompatibility of human skin cell seeded in the scaffold, MTS assay was used to test the cell viability. Compared to the original amount of cells seeded on 24 well plates directly, FBs seeded in the scaffold presented about 80% attachment ratio (data not shown). The FBs viable cell numbers on sponge-like scaffolds from day 1 to day 14 were measured (Figure 4A). 14 days post-seeding culture, the cell density of FBs grew within the scaffold and demonstrated a noteworthy enhancement, indicating that our scaffold had advantages for cell proliferation, differentiation and survivability. From SEM images of the scaffold (Figure 1C), we distinctly observed homogeneous and smooth structures with open macro-porous surfaces within the scaffold. Figure 4B showed the photo of the FBs treated-scaffold at the 14^th^ day, on the contrary, the pore wall surfaces of the cell-treatment scaffold were characterized to be rough and fractured, and presumably degraded by FBs.

### *In vitro* mono-cell culture and fluorescent studies

**Figure 5:**
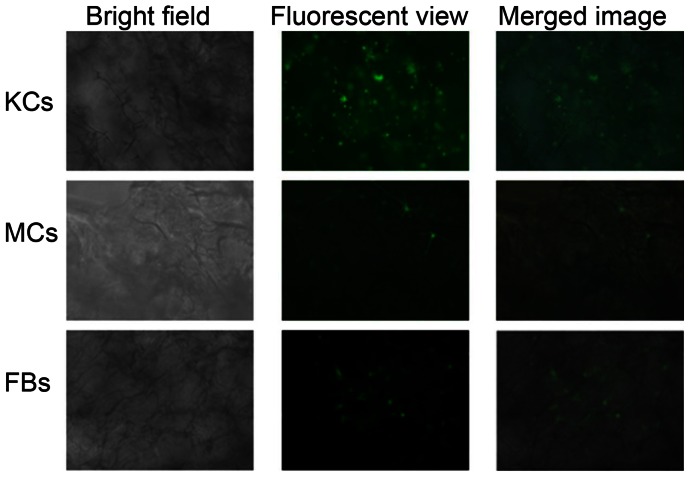
Photographed human KCs, MCs and FBs cultured in the scaffold on bright field, fluorescent and merged phase. Fluorescent compound, PKH-67 (green), was used to stain cells.

The cultures of human KCs, MCs and FBs in collagen/HA/gelatin scaffold were observed under fluorescent microscope after fluorescence staining, respectively. To detect the skin cell distribution, we used PKH67 to stain the cells before seeding in the scaffold (Figure 5). Three kinds of cells were all normally proliferated under the existence of collagen/HA/gelatin scaffold which were proven to have benefits for cell growth as we mentioned before. Our scaffold was verified having appropriate pore size and water absorption ability for human KCs, MCs and FBs growth (Figure 2 and 3).

### Immunofluorescence of the skin equivalent paraffin section

**Figure 6:**
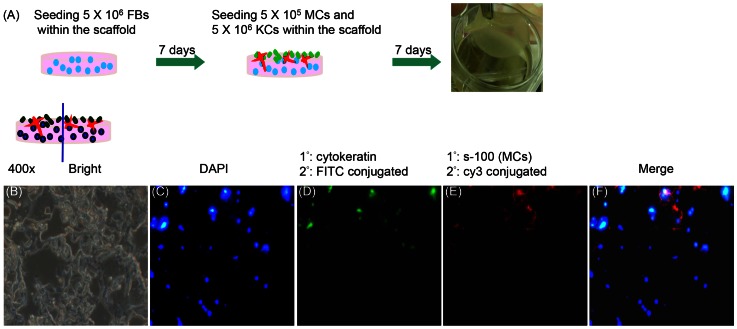
The protocols and fluorescent photos of 3D human skin equivalent. (A) The protocols of 3D human skin equivalent. (B) Paraffin section of the 3D human skin equivalent under microscope in bright view (400×). (C–E) Fluorescent images of KCs, MCs, and FBs cultured in scaffold for 14 days, and were stained with DAPI (blue); anti-cytokeratin to mark KCs (green); anti-s-100 for MCs (red). (F) The merged image was of KCs, MCs, and FBs together. Arrows pointed to KCs, MCs, and FBs with specific colors.

To produce a model which simulated the real human skin physiological conditions, we continually constructed a co-culture system for 3D human skin equivalent (Figure 6A). The skin model was generated by seeding KCs and MCs on the scaffold with FBs seeded on for 7 days in advance. After incubating co-cultured cells for another 7 days, samples were vertically sectioned, stained with immunofluorescence reagents and observed under the fluorescent microscope (400×). Figure 6B was the paraffin section image of the skin model under the bright field. Figure 6C showed all cell nucleus counterstained with DAPI in blue color. In Figure 6D, KCs were marked with anti-cytokeratin followed by FITC conjugated secondary antibody to present a green fluorescent color. MCs were labeled with anti-s100 protein, the secondary antibody conjugated with cy3, to express a red color (Figure 6E). From the immunoflurescent image, the cells stained with DAPI but not tagged with anti-cytokeratin or anti-s100 protein were FBs. We merged the images of Figure 6C–E together to perform in Figure 6F and found that epidermal KCs and MCs distributed above while dermal FBs appeared in the bottom part.

### The collagen quantification

**Figure 7:**
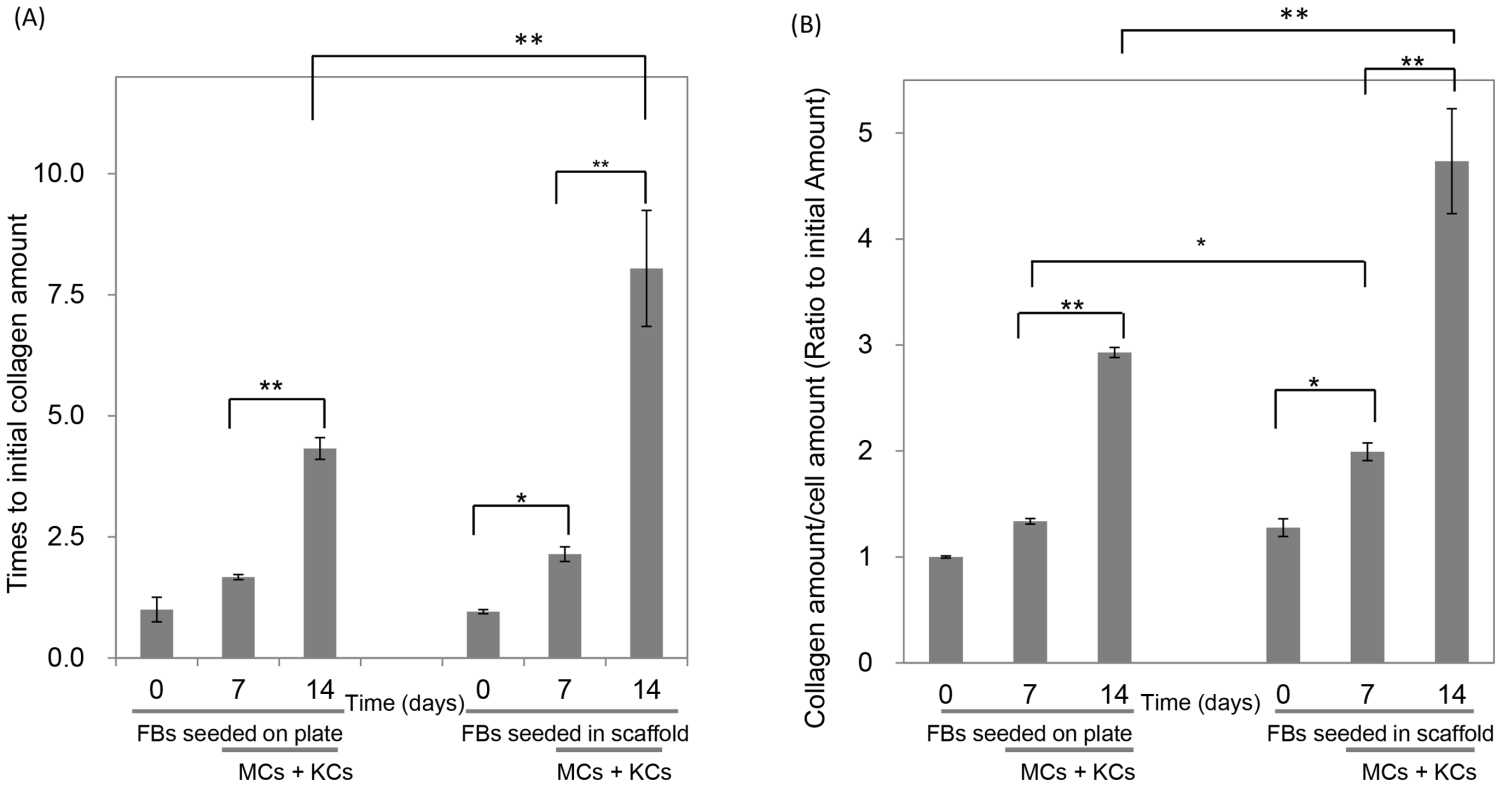
Collagen amount secreted from cells seeded on plate or in the scaffold. On the plate or in the scaffold, FBs raised for the first 7 days, after that, KCs and MCs seeded in for another 7 days. (A), the total collagen amounts on the plate or in the scaffold were shown. (B), the specific collagen amount measured with dividing collagen amount by cell amount.

Sirius Red dye was applied to measure the collagen production by three co-culture cells on the plate well or in the scaffold. We quantified the original quantity within the scaffold as the basal level since collagen is one of raw materials. Figure 7A showed the total collagen amount on the bio-supporting scaffold and it was observed that on the plate, after seeding MCs and KCs with FBs the amount was obviously increased on the 14^th^ day. In the scaffold, after FBs seeded for 7 days, collagen visibly increased, we further seeded MCs and KCs for another 7 days and it increased considerably higher than on the plate. The initial total cell amount on the 7^th^ or 14^th^ day was also measured and after 7 days, FBs apparently increased; on the 14^th^ day, with three kinds of cells co-culture, the cell number enlarged (data not shown). Figure 7B demonstrated the specific collagen amount measured by dividing preceding data by the cell amount and illustrated that on the 7^th^ day, the specific collagen amount in the scaffold was notably higher than on the plate. On the 14^th^ day, compared to collagen yielded by cells seeded on the plate, the specific collagen production in the scaffold was about 1.6 times more.

### Evaluation of the wound size

**Figure 8:**
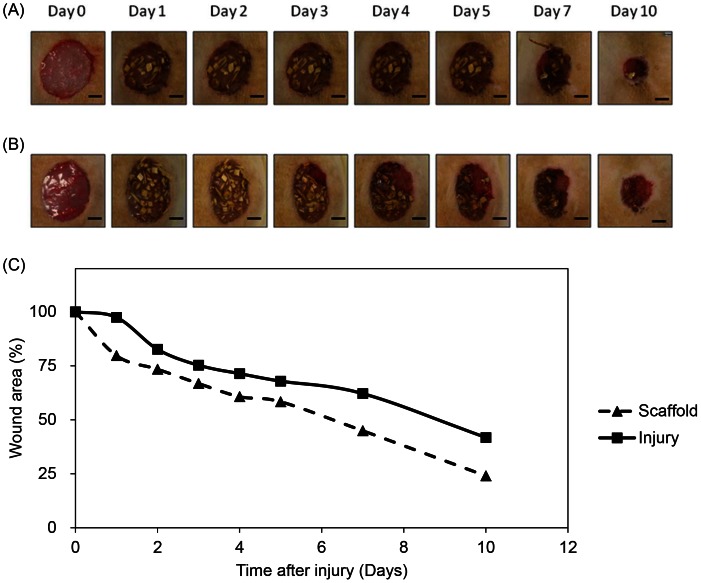
The healing pattern of the wounds in different conditions. (A) Scaffold treated and (B) injury wound after 0, 1, 2, 3, 4, 5, 7 and 10 days after injury. The wound healing efficacy of the scaffold was evaluated in a full thickness wound model. Following anaesthetized a full thickness excisions of 2 cm in diameter were created by a surgical knife of male Wistar rats. For treatment group after excision was made, the scaffold was covered on the wound immediately. For injury group wounds were not covered for comparison. From the first day after injury, the healing of wound from injury group was slower than scaffold treated wound until 10 days after injury. Scale bar  = 0.5 cm. (C) Wound contraction ratios of scaffold and injury at different times. By examining the wound area at definite days, the reduction of wound area was calculated. The surface area of the excisional wounds was calculated as described in methods. The wound area decreased rapidly in the presence of scaffold when compared with the control since first day after injury. The wound area in control group was 60% of the original size on day 7. This percentage was reached almost 3 days earlier at scaffold group. The difference between wounds of injury and scaffold group were statistically significant at day 10. Data are presented as the mean ± standard error.

The wound healing efficiency of the scaffold was evaluated via an *in vivo* full thickness wound model and quantified with excision wound area image analysis. The wound areas of both injury and treatment group shrank with time and were shown in Figure 8. Wound area of treatment group at day 1, 2, 3, 4, 5, 7 and 10 after injury was 79.7±3.4%, 73.4±3.5%, 66.8±2.2%, 60.7±5.0%, 58.3±6.1%, 44.9±4.3% and 24.0±2.1%, respectively, which are all smaller than wound area of the injury group (97.4±5.5%, 82.7±2.2%, 75.3±3.7%, 71.4±3.8%, 67.9±8.0%, 62.1±9.4% and 41.8±5.3%). From the beginning, the wound area of the treatment group was smaller than the injury group, and this repair trend was consistent 10 days after injury. The scaffold-treated wound healing achieved more than 50% wound closure after 7 days and up to 75% wound closure within 10 days. This exhibited that the *in vivo* wound shrinking rate of the treatment group was faster and the curing ability was greater.

### *In vivo* histological study

**Figure 9:**
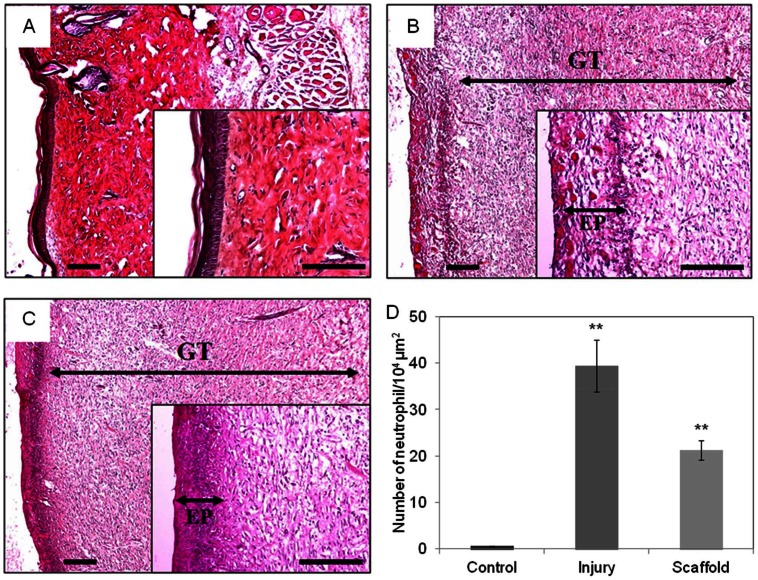
H&E stained sections for the morphological evaluation of skin wounds. Ten days after injury, rats were scarified, wound skin was fixing in 4% of paraformaldehyde. The skin was stained with H&E for histological observation. Ten randomly selected areas of dermis from each sample were examined at a magnification of 400× for counting neutrophil. Scaffold group (A), injury group (B) and control (C) wounds at 10 days after injury. Both scaffold and injury group wounds have granulation tissue. The epidermis of treatment group was denser than injury epidermis. Wounds of treatment group were had less neutrophil infiltrated compare to injury group (D). Scale bar  = 200 μm. EP, epithelial layer, GT, granulation tissue.

Histological exam of the H&E stained skin section showed the granulation tissues of the control group (without injury, Figure 9A), the group without injury (Figure 9B) and the group with scaffold treatment (Figure 9C). The epidermis in the scaffold group was denser than the injury group, and proved that the skin repairing was facilitated by the scaffold. During the wound healing process, neutrophils secrete substances to accelerate KCs differentiation and to delay wound closure. Compared to the injury group, and there was less neutrophil infiltration in the scaffold treatment group (Figure 9D). By applying this scaffold, neutrophil infiltration would be decreased and wound closure would be more rapid. These evidences, including the improved healing speed, the increased epidermis density and reduced neutrophil infiltration, suggested that this scaffold is suitable for excision wound healing.

## Discussion

To construct a proper scaffold in skin tissue engineering, several crucial factors to consider include pore size range, mechanical strength, biodegradability and *in vivo* accommodation. Cross-linking has been confirmed to play an important role related to the scaffold porous structure distribution and water containing ability. For this research, the collagen/HA/gelatin scaffold was produced through cross-linking and lyophilization (Figure 1D). After cross-linking, this scaffold avoided dissolving into water and was able to retain the culture medium. In comparison to the present commercial samples used for surgery or dermatology dressings, the scaffold exhibited a much higher capacity for water absorption (Figure 2). The swelling ratio results revealed that the scaffold possessed excellent porous lamellar matrix spaces which increased the water containing capacity. Because of the high water absorption feature, the sponge-like matrices were optimal for cells to culture in [39], [40]

The degradation data presented divergences for different test-enzymes including lysozyme, hyaluronidase and collagenase I (Figure 3). At short time intervals (by hours) or with low lysozyme concentrations (<2,000 U/ml), we did not observe obvious weight loss of the scaffold (data not shown). Our scaffold was composed by either *N*-acetylmuramic acid or *N*-acetyl-D-glucosamine residues, which explained why lysozyme hardly hydrolyzed the scaffold since lysozyme has a similar catalytic mechanism to hyaluronidase. We observed that both enzymes had high correlation biodegradation results. In this scaffold, collagen was easily degraded by collagenase and after it had decomposed, the scaffold lost its structural properties and dissolved in 2 h.

When creating the skin graft, the development of the dermis over the model was apparently accelerated by the application of skin cells to the graft [41]. FBs play active roles in a variety of biological processes such as the production of collagen, HA and ECM proteins. In particular, FBs generate intra/extracellular cytoskeleton tension forces which allow for interaction with the ECM [42]. Fluorescent observations showed the three skin cells, FBs, MCs and KCs, seeded in the scaffold separately and demonstrated that they proliferated normally, confirming the benefit of these materials to cell growth (Figure 5).

The advantage of this human skin scaffold is that it harbors various cell types (KCs-epidermis, MCs-epidermis, FBs-dermis) surrounded by a local microenvironment, resembling the i*n vivo* situation similar to human normal skin compared to conventional mono/bilayer cultures. The interconnected pores within the scaffold provided the opportunity for interactions of biological cytokines and growth factors released from KCs, MCs and FBs [43], [44]. Due to the physiological interactions and mutual communication between these three kinds of cells, co-culturing them sequentially allowed them to grow in a physiological-like condition within the scaffold [45], [46]. This model mimicked the human skin structure allowing the successful formation of a human skin equivalent (Figure 6). In addition, our scaffold served as exogenous biomaterial that was seeded in the bottom with FBs in order to provide a microenvironment permissive of skin cell formations, which thereby increased the required bionic characteristics [35], [39].

Collagen is an important component for cell proliferations and tissue body formation which are dependent on FB production [22]. Therefore, in skin wound healing promotion, detecting the concentration of the amount of collagen secreted is a key index. After seeding FBs for 7 days, MCs and KCs were added and seeded for another 7 days, and the collagen amount elevated dramatically, demonstrating that the co-culture of FBs combined with mixed MCs and KCs increased the collagen quantity (Figure 7A). This exhibited the importance of a co-culture model in creating the physiological environment. In Figure 7B, similarly, KCs and MCs were added to the co-culture with FBs. Dividing the collagen amount by the cell amount allowed for comparison of the specific collagen production of individual cells which showed that the collagen was increased more significantly on the scaffold than on the plate. The resulting data from the scaffold was notably higher than the plate on the 7^th^ day, which exhibited that not only the cell proliferation was promoted, but the individual collagen manufacturing abilities were also enhanced. Since our scaffold has demonstrated the ability to promote collagen secretion, it is potentially a good biomaterial for wound healing in tissue engineering.

The progress of wound healing can be categorized into three overlapping stages: inflammation, cell proliferation and tissue remodeling [47], [48]. In the inflammation stage, many immune cells such as neutrophils, monocytes and lymphocytes are abundant around the wound in order to eliminate pathogens. Histological analysis showed that when the skin has an excision wound, the neutrophils infiltrated the wound area (Figure 8), which is a phenomenon that is consistent with previous studies [37], [49], [50]. Previous studies also demonstrated that excess neutrophils reduced the wound closure rate [51], [52] and had damage to normal cells [53], [54]. The *in vivo* results above revealed that this scaffold assisted and accelerated wound closure rate by reducing neutrophils and the over-inflammation response. As the neutrophil numbers begin to decline, macrophages take over and refill the wound site. At the same time, re-epithelialization begins with the migration of epithelial cells from the surrounding epidermis at the wound edge, which occurs with KC migration across the granulation tissue from deep within the dermis and basal cells of the wound edge [37]. The most crucial function of the epidermis is to prevent water loss, and rapid re-epithelialization accomplishes this in addition to reducing the time of exposure to potential infections [55], [56]. In this study, granulation tissue, which contained FBs, collagen and blood vessels, was observed in both the scaffold group and injury group but not in the control group, indicating that both groups healed normally and that the scaffold did not interrupt the wound healing process. 10 days after injury, the epidermis tissue density of the treatment group was high in the no-dressing group, which suggested that more mature epithelium grew. The earlier wound closure and denser epidermis indicated that applying this scaffold caused less water loss and bacterial infection during wound healing procedures. This scaffold potentially accelerated the healing process by inhibiting the infiltration of neutrophils and promoting re-epithelialization.

Our collagen/HA/gelatin scaffold is with great potential due to the suitable pore size, the high swelling ratio, the good cytocompatibility, and the collagen production enhancement. The skin pharmaceutical and therapeutic wound care dressing market is a significant segment. Bio-functions of traditional dressings in the past are just for keeping wound dry and preventing microorganism infection. In clinical applications, we know that moist and warm surroundings facilitate the skin wound repairing. Effective scaffold shall consider several important factors including skin tissue assessments/tissue deficit managements, moisture containing balances, infection preventions, inflammation controls and dermatological wound edge progression enhancings. There are other issues needed to be discussed, such as the patient healthy conditions (e.g. diabetes), the injury type being created by physical or chemical damage, and the social/environmental properties. We will keep focusing on these important subjects about skin wound dressings in future studies.

## Conclusions

We found that the sponge-like collagen/HA/gelatin scaffold provided an optimal pore size and water absorption for human skin cell growth. This scaffold could be cleaved by lysozyme, hyaluronidase and collagenase I, which demonstrates its biodegradability. Fluorescent staining presented the FBs, MCs and KCs co-culturing in the scaffold which was able to mimic the human epidermis and dermis structures. The scaffold helped the wound-close faster with no further inflammation or other side effects. Overall, the results from *in vitro* and *in vivo* studies suggested that this scaffold is a potent matrix for skin regeneration.
