# Correction: Novel Biodegradable Porous Scaffold Applied to Skin Regeneration

**DOI:** 10.1371/annotation/4d5ef06d-b800-4d0c-b809-d3cb7a5d00c6

**Published:** 2013-11-04

**Authors:** Hui-Min Wang, Yi-Ting Chou, Zhi-Hong Wen, Chau-Zen Wang, Chun-Hong Chen, Mei-Ling Ho

The name of the fourth author was incorrect.

The correct name is: Chau-Zen Wang

The correct version of the citation is: Wang H-M, Chou Y-T, Wen Z-H, Wang C-Z, Chen C-H, et al. (2013) Novel Biodegradable Porous Scaffold Applied to Skin Regeneration. PLoS ONE 8(6): e56330. doi:10.1371/journal.pone.0056330

The correct version of the Author Contributions is: Conceived and designed the experiments: HMW YTC ZHW CHC. Performed the experiments: HMW YTC ZHW CHC. Analyzed the data: HMW YTC ZHW CZW CHC MLH. Contributed reagents/materials/analysis tools: HMW YTC ZHW CHC. Wrote the paper: HMW YTC ZHW CZW CHC MLH. 

